# The Uncertain Relationship between Air Pollution and Risk of Preterm Birth: Does Spatial Variation Drive Disparate Findings?

**DOI:** 10.1289/ehp.124-A147

**Published:** 2016-08-01

**Authors:** Nate Seltenrich

**Affiliations:** Nate Seltenrich covers science and the environment from Petaluma, CA. His work has appeared in *High Country News*, *Sierra*, *Yale Environment 360*, *Earth Island Journal*, and other regional and national publications.

Air pollution isn’t just bad for those who breathe it; it also could potentially impact the future health of unborn babies. For instance, maternal exposures to fine particulate matter (PM_2.5_) and nitrogen dioxide (NO_2_) have been associated with small but consistent decreases in birth weight.^1^ ^,^ ^2^ ^,^ ^3^ However, associations with preterm birth have been less consistent. A new study of more than 258,000 New York City births published this month in *EHP* takes an innovative approach to this question by considering the hospital where a baby is born as a factor that may explain the results of studies of air pollution and preterm birth.^4^

Biologically speaking, there’s some reason to believe air pollution could somehow contribute to preterm birth, says study coauthor David Savitz, a professor of epidemiology, obstetrics, and gynecology at Brown University. “There’s not a compelling case that [an association] should be there but a plausible explanation if it were,” he says, citing inflammation and oxidative stress as potential mechanisms. This question is important because preterm birth is associated with infant mortality and with disease in childhood and possibly into adulthood.^5^ ^,^ ^6^ If air pollution were a causative factor, then better regulation of air pollution could help prevent preterm births.

**Figure:**
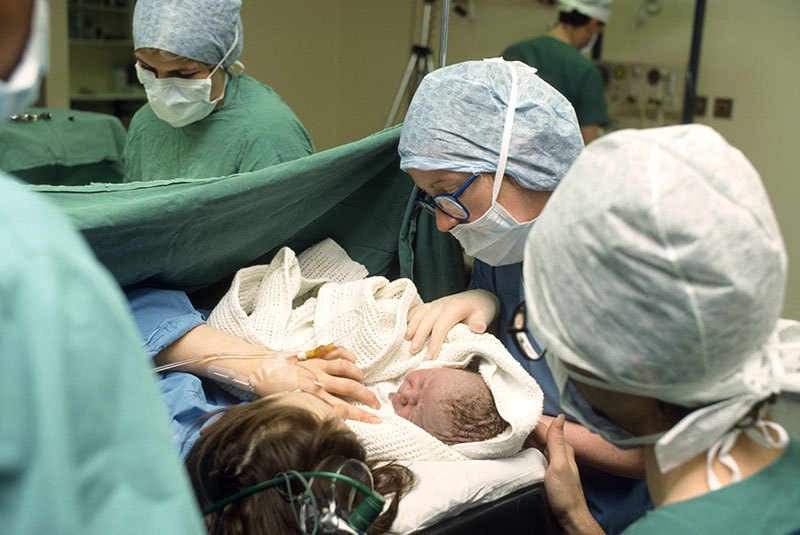
Controlling for the rate at which hospitals perform birth-related medical interventions could help researchers settle the question of whether air pollutants contribute to preterm birth. © Sally and Richard Greenhill/Alamy Stock Photo

Previous studies have been divided on whether air pollution contributes to preterm birth, with some showing a modest positive association, some a negative association, and others no association at all.^1^ ^,^ ^3^ Part of the reason that results are so disparate is that preterm birth is a complex outcome to study, potentially influenced by a wide range of factors.^7^

The authors used data from the New York City Community Air Survey^8^ and from regulatory air pollution monitors to estimate each mother’s exposure to PM_2.5_ and NO_2_ during pregnancy, based on her address at delivery. To evaluate birth characteristics, they used both birth certificates and hospital records. This allowed them to separate preterm births that occurred spontaneously from those initiated by a medical intervention like cesarean delivery or induced labor. With non-spontaneous births, researchers have no way of knowing whether the pregnancy would have otherwise ended in a term or preterm birth.

“Medical interventions that affect the timing of birth vary very, very widely [in terms of the rates at which they are used],” says senior author Thomas Matte of the New York City Department of Health and Mental Hygiene. “Focusing on spontaneous preterm birth is one way of trying to eliminate that factor from the results.”

Another way, he says, is to control for the birth-intervention rate of individual hospitals, a novel feature of this study. Since the rates of interventions vary among New York City hospitals, failure to account for this variable could affect how preterm birth rates appear to relate to mothers’ pollution exposure levels. Indeed, the researchers discovered that mothers with higher pollution exposures tended to deliver at hospitals with lower intervention rates.^4^

Ultimately, the researchers uncovered no relationship between PM_2.5_ and preterm birth, and a modest decrease in preterm birth associated with higher exposures to NO_2_, suggesting that NO_2_ was somehow protective.^4^ But when they controlled for hospital of birth, the negative association was diminished, indicating that future studies should assess spatial and temporal components of exposure separately. The researchers concede that because a protective effect of NO_2_ for preterm birth seems biologically implausible, the negative association might be a consequence of any number of confounders or variables unaccounted for by their study.

“I think it’s possible that there could be no true association between air pollution and preterm birth,” says Dave Stieb, a public health physician and epidemiologist with Health Canada. “But because of some of the factors that have been identified [in this study] as potentially confounding the associations observed here, we can’t really conclude that yet,” he says. “It highlights the need for more thoughtfully designed studies like this one, where there is a rich body of exposure data and outcome data.” Stieb was not involved with the study.

Ulrike Gehring at Utrecht University in the Netherlands is helping lead an ongoing study of 74,000 European women that also will investigate the link between air pollution and preterm birth (although the impact of hospital practices will not be assessed).^9^ Results to date have not been particularly compelling, she says. Future work could target regions with substantially higher pollution levels or different pollutant mixtures, Gehring says, such as cities in India or China with poor outdoor air or rural areas where indoor burning of biomass is prevalent.
